# Sequential Linker Installation in Metal–Organic Frameworks

**DOI:** 10.1021/acs.accounts.4c00564

**Published:** 2024-10-16

**Authors:** Zongsu Han, Yihao Yang, Joshua Rushlow, Rong-Ran Liang, Hong-Cai Zhou

**Affiliations:** Department of Chemistry, Texas A&M University, College Station, Texas 77843, United States

## Abstract

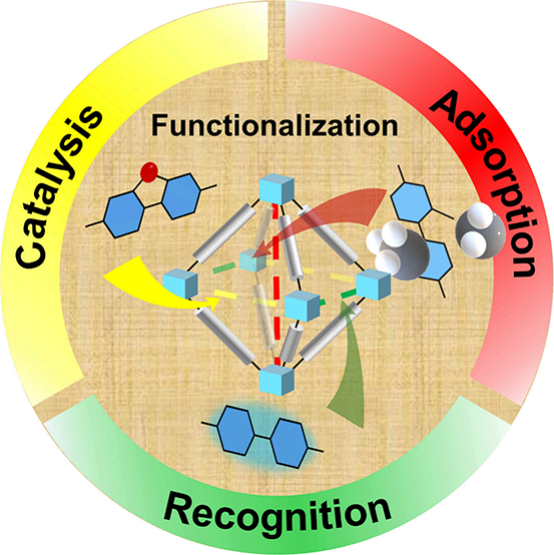

Metal–organic frameworks (MOFs) represent a sophisticated blend of inorganic and organic components, promoting the development of coordination chemistry greatly and offering a versatile platform for tailored functionalities. By combining various metal nodes, organic linkers, and functional guests, MOFs provide numerous pathways for their design, synthesis, and customization. Among these, sequential linker installation (SLI) stands out as a novel and crucial strategy, enabling the precise integration of desired properties and functions at the atomic scale. SLI enhances structural diversity and stability while facilitating the meticulous construction of robust frameworks by leveraging open metal sites and functional organic linkers at targeted locations. Compared to the direct synthesis of MOFs, postsynthetic modification methods allow for precise regulation of their structures and corresponding properties. While unlike conventional postsynthetic modification methods, SLI requires the careful selection of linkers and framework design to ensure precise positioning for installation, which gives rise to the well-designed and ordered positions for the installed linkers, confirmed directly by X-ray diffraction technology.

Recent advancements in MOF synthesis have led to the creation of increasingly tailored flexible matrix structures, particularly due to the diverse connection modes of multicore metal clusters, especially for the Zr_6_ cluster. The spatial hindrance of certain ligands has resulted in the formation of unsaturated metal clusters and various missing linker pockets. Examples of these advanced MOFs include PCN-606, PCN-608, PCN-609, PCN-700, and PCN-808, which feature specific open metal sites and certain framework flexibility conducive to SLI. Strategically positioned open metal sites within these frameworks serve as predetermined anchor points for desired functional molecules, while the frameworks’ flexibility can accommodate molecules of varying sizes to a certain extent, enlarging the scopes of application greatly. This precise positioning of functional groups enables the creation of tailored sites for enhanced applications, such as adsorption, catalysis, and recognition.

In this Account, we delve into the intricate process of designing and synthesizing MOFs with appropriate missing-linker pockets for the aforementioned applications. We discuss the meticulous selection of functional linkers and the methods used to insert them into the corresponding missing-linker pockets within the MOFs. Additionally, we explore the diverse properties and functionalities of the resulting MOFs, focusing on their adsorptive, catalytic, and recognition performance. Furthermore, we provide insights into the future trajectory of SLI methods, complemented by our recent works. This Account not only reviews the evolution of the SLI method but also underscores its practical applications across various functional domains, paving a rational pathway for the future development of advanced multifunctional MOFs through this method.

## Key References

- YuanS.; LuW.; ChenY.-P.; ZhangQ.; LiuT.-F.; FengD.; WangX.; QinJ.; ZhouH.-C.Sequential Linker Installation: Precise Placement of Functional Groups in Multivariate Metal–Organic Frameworks. J. Am. Chem. Soc.2015, 137, 3177–318025714137 10.1021/ja512762r.^1^*The first sample for the sequential linker installation toward MOF, which constructs a series of MOFs with similar skeleton structures and scalable networks*.
- YuanS.; ChenY.-P.; QinJ.-S.; LuW.; ZouL.; ZhangQ.; WangX.; SunX.; ZhouH.-C.Linker installation: engineering pore environment with precisely placed functionalities in zirconium MOFs. J. Am. Chem. Soc.2016, 138, 8912–891927345035 10.1021/jacs.6b04501.^2^*Systematic study of a series of ligands with various sizes and shapes for the linker installation toward MOF*.
- PangJ.; YuanS.; QinJ.-S.; LollarC. T.; HuangN.; LiJ.; WangQ.; WuM.; YuanD.; HongM.; ZhouH.-C.Tuning the ionicity of stable metal-organic frameworks through ionic linker installation. J. Am. Chem. Soc.2019, 141, 3129–313630689379 10.1021/jacs.8b12530.^3^*Ionicity of MOFs can be regulated through the linker installation by different linkers, with enhanced adsorption properties*.
- HanZ.; SunT.; LiangR.; GuoY.; YangY.; WangM.; MaoY.; TaylorP. R.; ShiW.; WangK.-Y.; ZhouH.-C.Chiral linker installation in a metal-organic framework for enantioselective luminescent sensing. J. Am. Chem. Soc.2024, 146, 15446–1545238776639 10.1021/jacs.4c03728PMC11157530.^4^*The first sample for the chiral linker installation, which can be used for the enantioselective recognition*.

## 1 Introduction

Metal–organic frameworks (MOFs) are a novel class of advanced materials that have garnered significant attention in coordination chemistry, materials science, and environmental engineering.^5−7^ These materials consist of metal ions or clusters coordinated to organic ligands, forming multidimensional structures with abundant space to accommodate various guest molecules. The unique aspect of MOFs lies in their highly ordered, porous, and regulated architectures, which can be precisely tuned for diverse applications. These include gas storage, separation, catalysis, and molecular recognition, making them suitable for various fields such as medical treatment, environmental protection, and pollutant control.^8−15^

**Figure 1:**
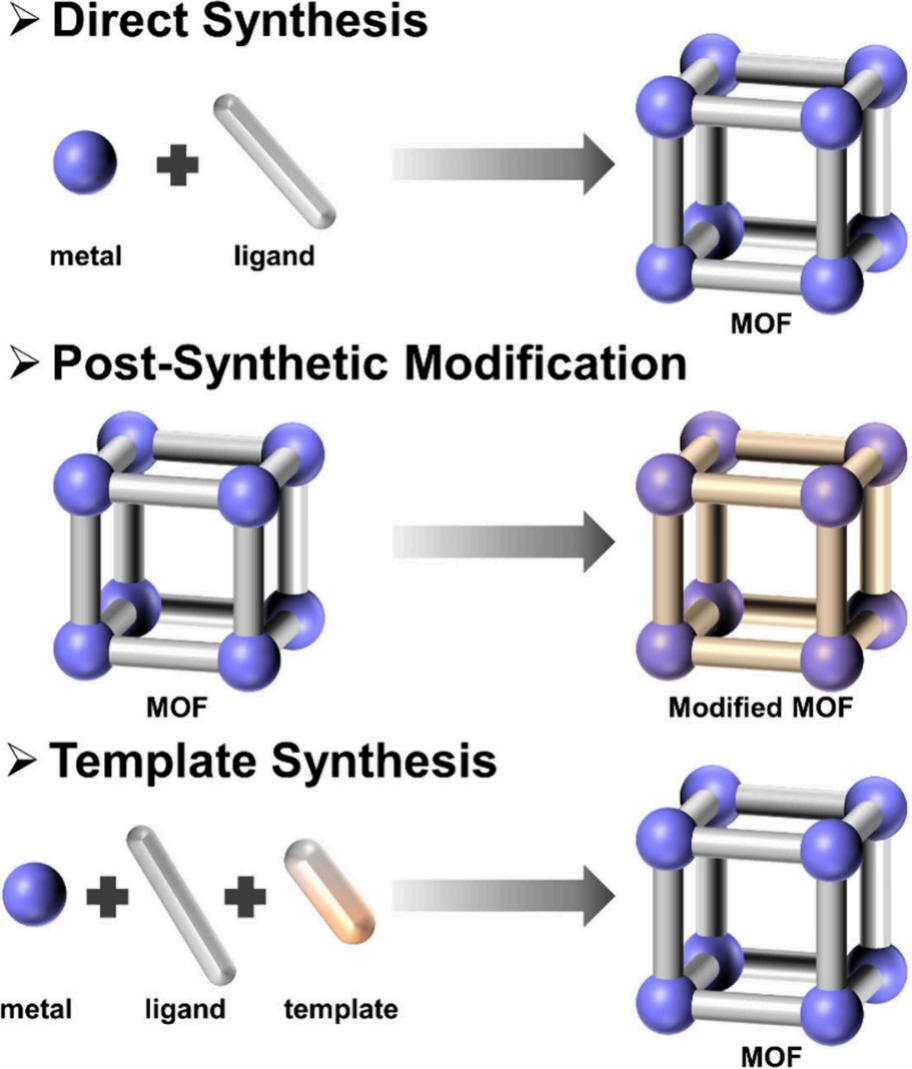
Classical construction methods of MOFs.

The construction methods of MOFs are fundamental to the development and application of these advanced materials. The methods mainly include direct synthesis, postsynthetic modification, and template synthesis (Figure 1). Direct synthesis is the most straightforward and widely used method for creating new MOFs, involving the combination of metal nodes with organic ligands under specific conditions to form the desired structures.^16−20^ Postsynthetic modification entails altering preformed MOFs to introduce new functionalities or improve their properties, providing an additional layer of control and allowing for fine-tuning after the initial synthesis.^21−25^ Template synthesis employs certain molecules as templates to guide the formation of MOF structures and morphologies, resulting in unique pore structures that are difficult to achieve through direct synthesis alone.^26−28^

**Figure 2:**
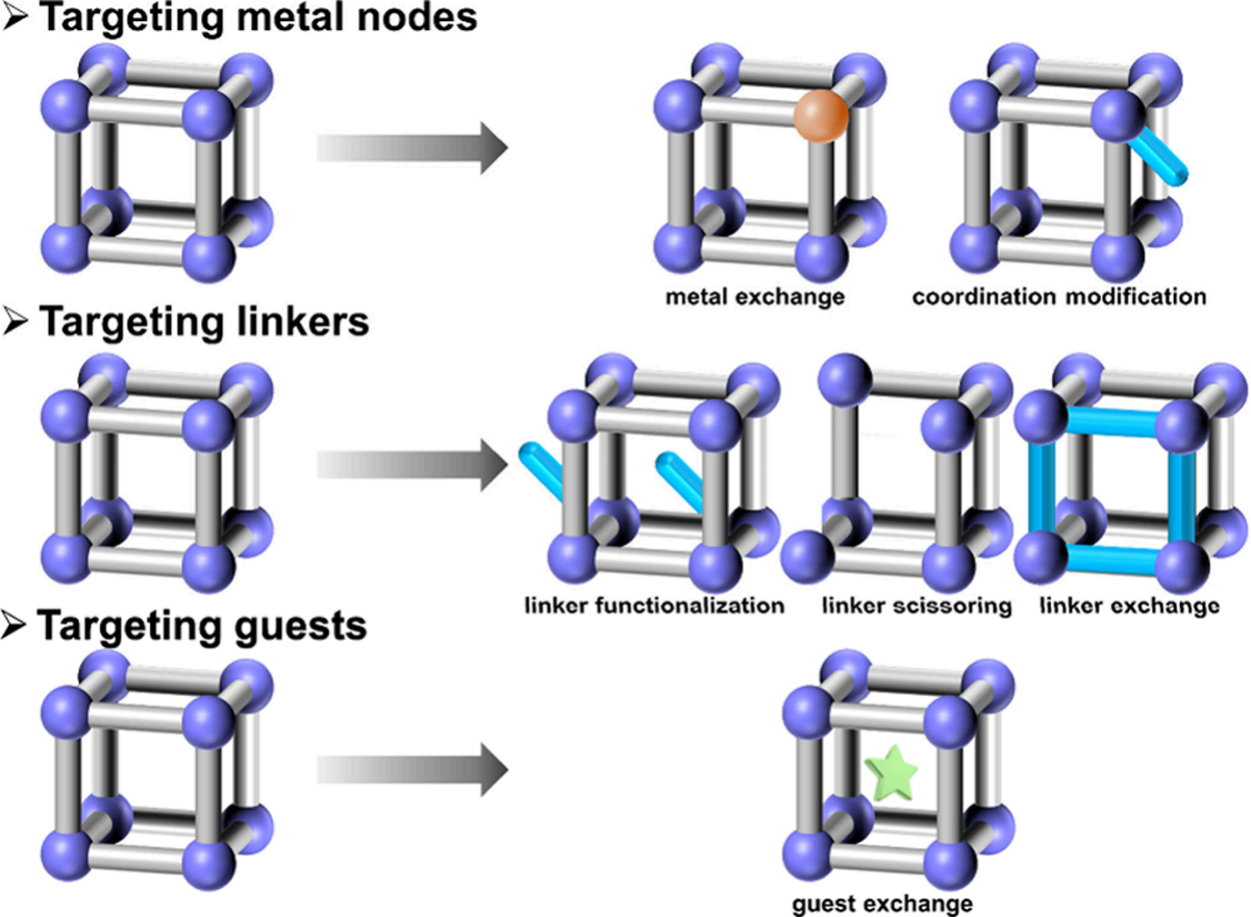
Classical postsynthetic modification methods toward MOFs.

Compared with direct synthesis and template synthesis, postsynthetic modification offers irreplaceable advantages by eliminating the need for numerous attempts to match metals, ligands, solvents, templates, and synthesis conditions, as well as mitigating the unpredictability of network structures and material properties. Postsynthetic modification allows for the decorations at three key positions (Figure 2): the metal nodes, the linkers, and the guests. This approach includes six main techniques: metal exchange^29,30^ and coordination modification^31−33^ targeting the metal nodes; linker functionalization,^34−36^ linker scissoring,^37,38^ and linker exchange^39,40^ targeting the linkers; and guest exchange^41−43^ targeting the guests.

Coordination modification offers distinct advantages over other postsynthetic modification methods by focusing on altering the coordination environment of the metal nodes. This approach provides precise and targeted modifications to the framework, typically resulting in minimal crystallinity loss, a common issue with other postsynthetic modification methods.^31−33^ Besides, this method enhances the functionality of MOFs while maintaining their structural integrity, making it a superior choice for advanced applications.

**Figure 3:**
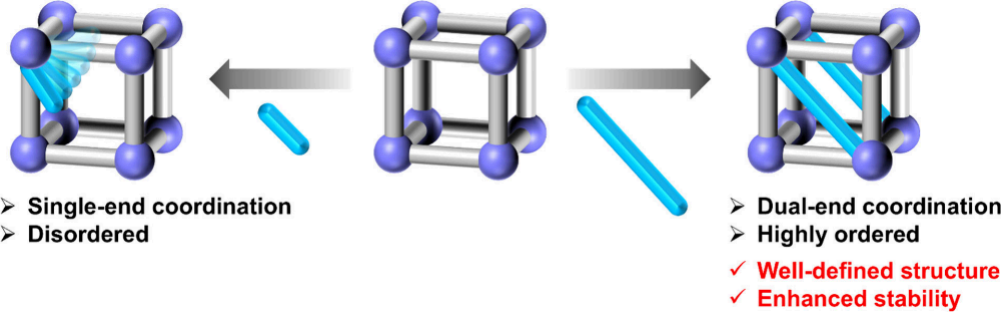
Comparisons of classical coordination modification (left) and sequential linker installation (right).

Sequential linker installation (SLI) is a specialized coordination modification method that requires dual-end coordination interactions, unlike the traditional single-end coordination in the coordination modification method between the framework and the modified molecule (Figure 3).^44−49^ This unique connection method demands precise matching of the installed molecule to the MOF, resulting in well-defined positions and structures that can be directly detected by X-ray diffraction technology and enhanced stability of the whole framework. A series of advanced characterization methods, particularly powder X-ray diffraction and neutron diffraction and scattering, can also be used for structure confirmation, which have also shown unique advantages to probe the structural insights. Furthermore, compared with the pore partition strategy^50,51^ which shares a similar design concept with the SLI method for targeted pore design, the SLI method emphasizes the ’sequence’ of a multistep installation process, which can introduce different linkers in order. This approach allows for the creation of structures that are difficult to achieve through one-pot synthesis. The precision in modification allows for enhanced control over the structures, properties, and functionalities of MOFs, making SLI a powerful technique in MOF synthesis and application.^52−57^

In this Account, drawing from our previous and recent works, we first focus on the design and selection of suitable frameworks to demonstrate the synthesis of commonly used MOFs for this method. We then discuss the selection of the installed linkers and the conditions for SLI. Following this, we explore the functionality changes that occur after SLI, particularly regarding catalysis, adsorption, and recognition functions. Finally, we present our suggestions and expectations for the future development of this method.

## 2 Design and Selection of Suitable Frameworks

SLI is a powerful method for introducing target linkers at specific positions, which generally require a framework with a certain degree of flexibility to provide enough space for insertion. The Zr_6_ cluster is frequently used due to its easily obtained structures, high-quality single crystals, abundant coordination sites, and various coordination modes. These characteristics facilitate direct structural analysis by single crystal X-ray diffraction and provide numerous potential open metal sites at various positions, making it an excellent host for the linker installation process.

**Figure 4:**
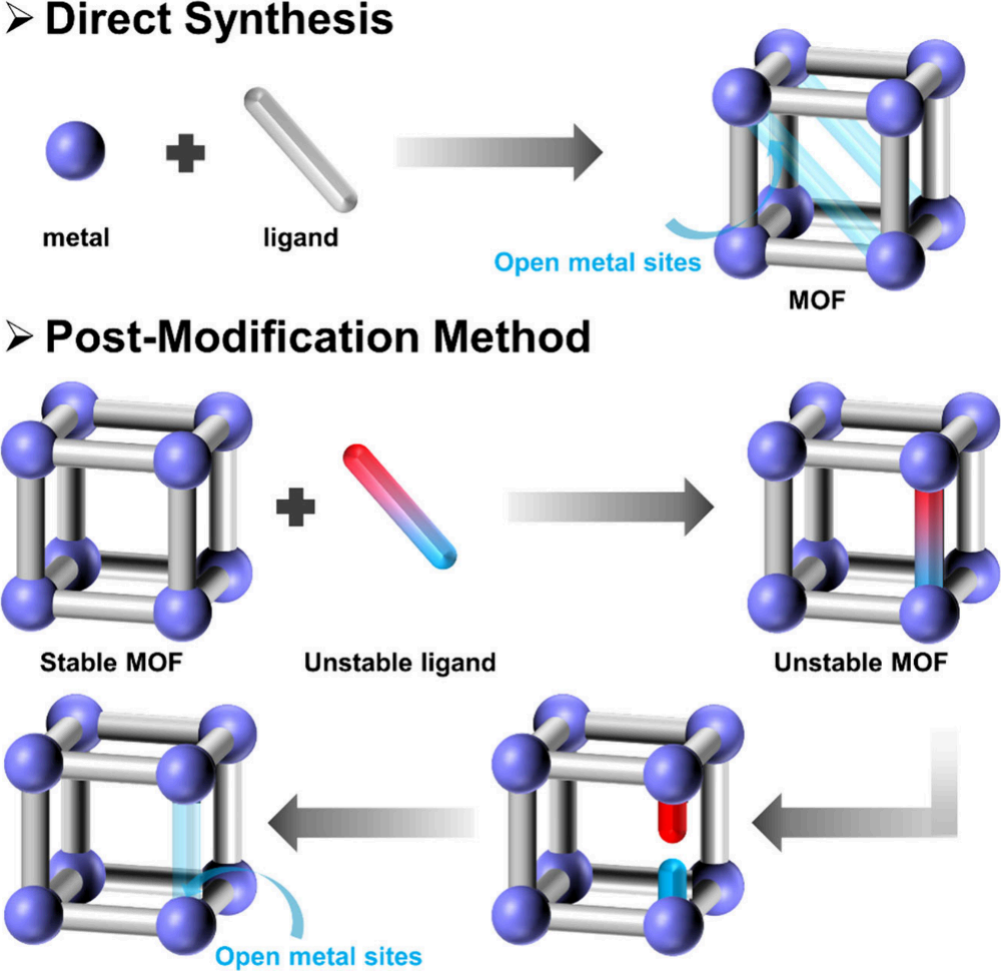
Classical pathways for the construction of suitable flexible MOFs for SLI.

There are two main pathways for constructing suitable flexible MOFs for SLI. The first is through direct synthesis using appropriate ligands. The second is a postmodification method, which usually involves a complex process, including linker exchange and linker scissoring, to break the coordination bonds between the metal clusters and the linkers, thus exposing the open metal sites for SLI (Figure 4).

**Figure 5:**
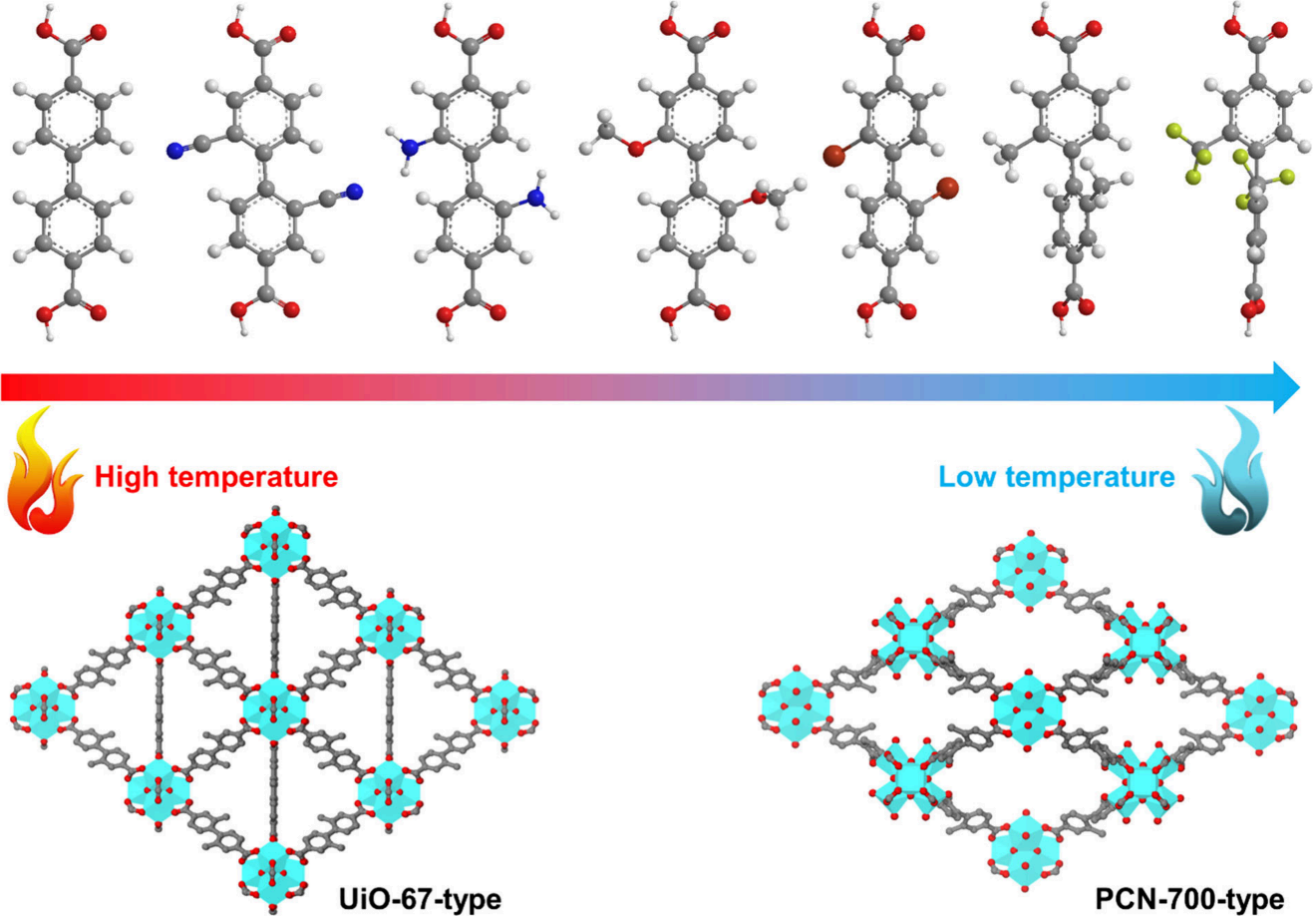
Constructions of UiO-67-type and PCN-700-type MOFs.

For direct synthesis, PCN-700-type MOFs have been the most extensively studied series, mainly by our group.^1−3,45,49,54^ The synthesis involves using unsaturated 8-connected Zr_6_ clusters and biphenyl-4,4′-dicarboxylate with 2- and 2′-substituent groups. A key challenge in synthesizing PCN-700 with unsaturated Zr_6_ clusters is the formation of competing phases, such as the UiO-67-type structure with saturated Zr_6_ clusters. From a thermodynamic perspective, the formation of the Zr-carboxylate bond is an exothermic process, making the saturated UiO-67 thermodynamically more favorable than the unsaturated PCN-700. From a kinetic perspective, in UiO-67-type structures, the two carboxylates of the linker adopt a coplanar conformation. In contrast, in PCN-700-type structures, the two carboxylates are perpendicular to each other, which necessitates the steric hindrance of 2- and 2′-bulky substituent groups on the ligand to force the phenyl rings and carboxylates into a perpendicular arrangement. Thus, the competitive formation of UiO-67-type and PCN-700-type structures can be viewed as a balance between thermodynamic and kinetic factors, with a dynamic steady state favoring the formation of the unsaturated structure (Figure 5).

**Figure 6:**
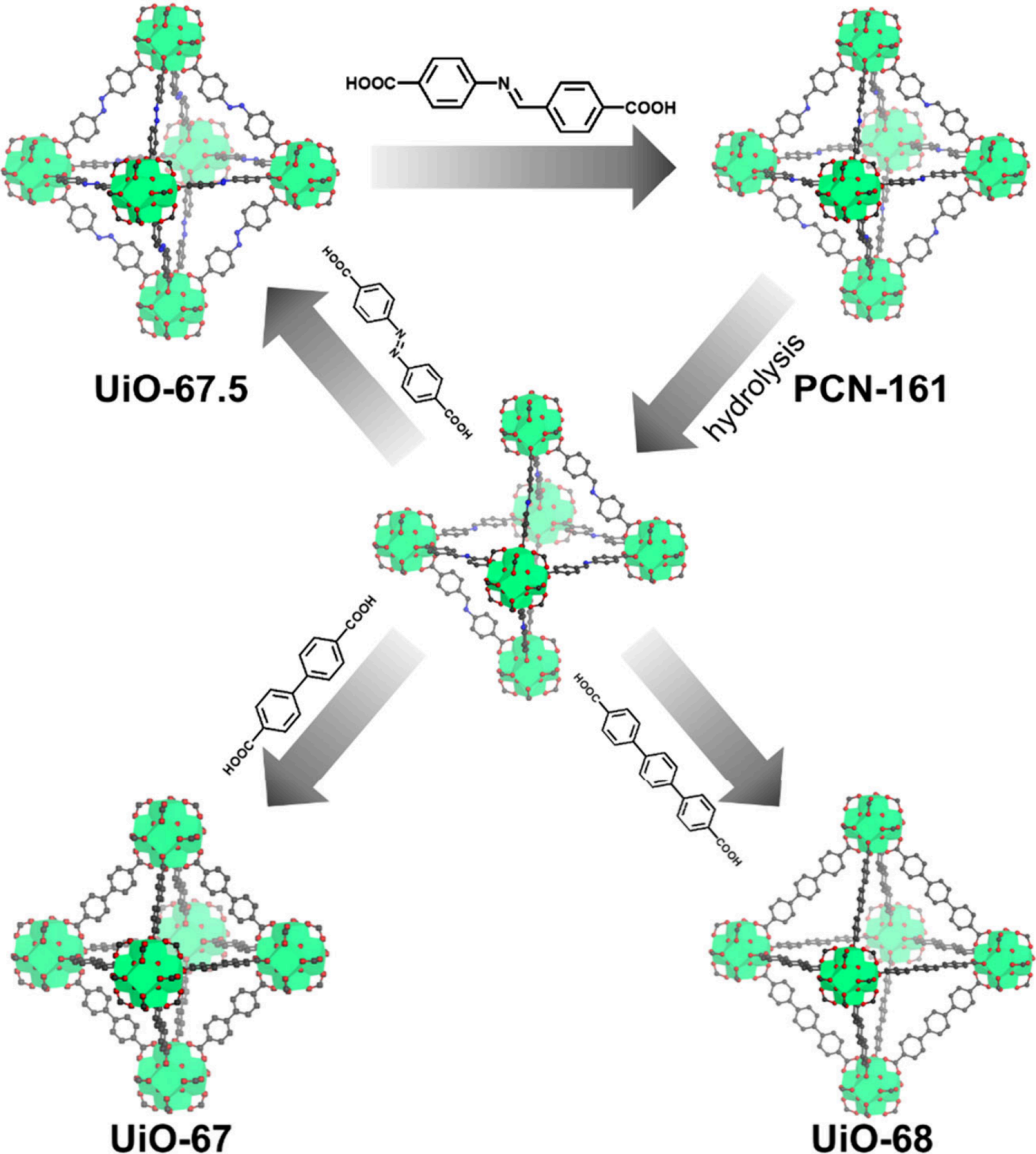
Constructions and transformations for PCN-161.

For the postmodification method, our group constructed a series of novel Zr_6_ cluster MOFs named PCN-16X (X = 1–4).^47^ These MOFs were obtained by linker exchange from the classical stable UiO-66-type MOFs, UiO-67.5, UiO-68, UiO-68.5, and UiO-69, respectively. The relatively unstable -C=N- bonds from the exchanged linkers were introduced into the UiO-66-type MOFs, transitioning them from robust to active. These -C=N- bonds were then easily broken by solvent to expose the open metal sites, facilitating further SLI processes to incorporate shorter or longer linkers, simultaneously (Figure 6).

In conclusion, the introduction of target linkers into MOFs at targeted positions through the SLI method is a powerful approach that necessitates a flexible framework to accommodate the insertion. The Zr_6_ cluster stands out as an ideal host due to its versatile coordination chemistry and easily analyzable single-crystal structures. Two primary pathways, direct synthesis and postmodification method, have been explored to create suitable flexible MOFs for SLI. Direct synthesis has been extensively studied, highlighting the challenges of balancing thermodynamic and kinetic factors to achieve the desired unsaturated structures. Postmodification methods leverage linker exchange to introduce flexibility and linker scissoring to expose open metal sites. Both approaches underscore the potential of SLI in advancing the design and functionality of MOFs, paving the way for innovative applications in material science.

## 3 Sequential Linker Installation Process

**Figure 7:**
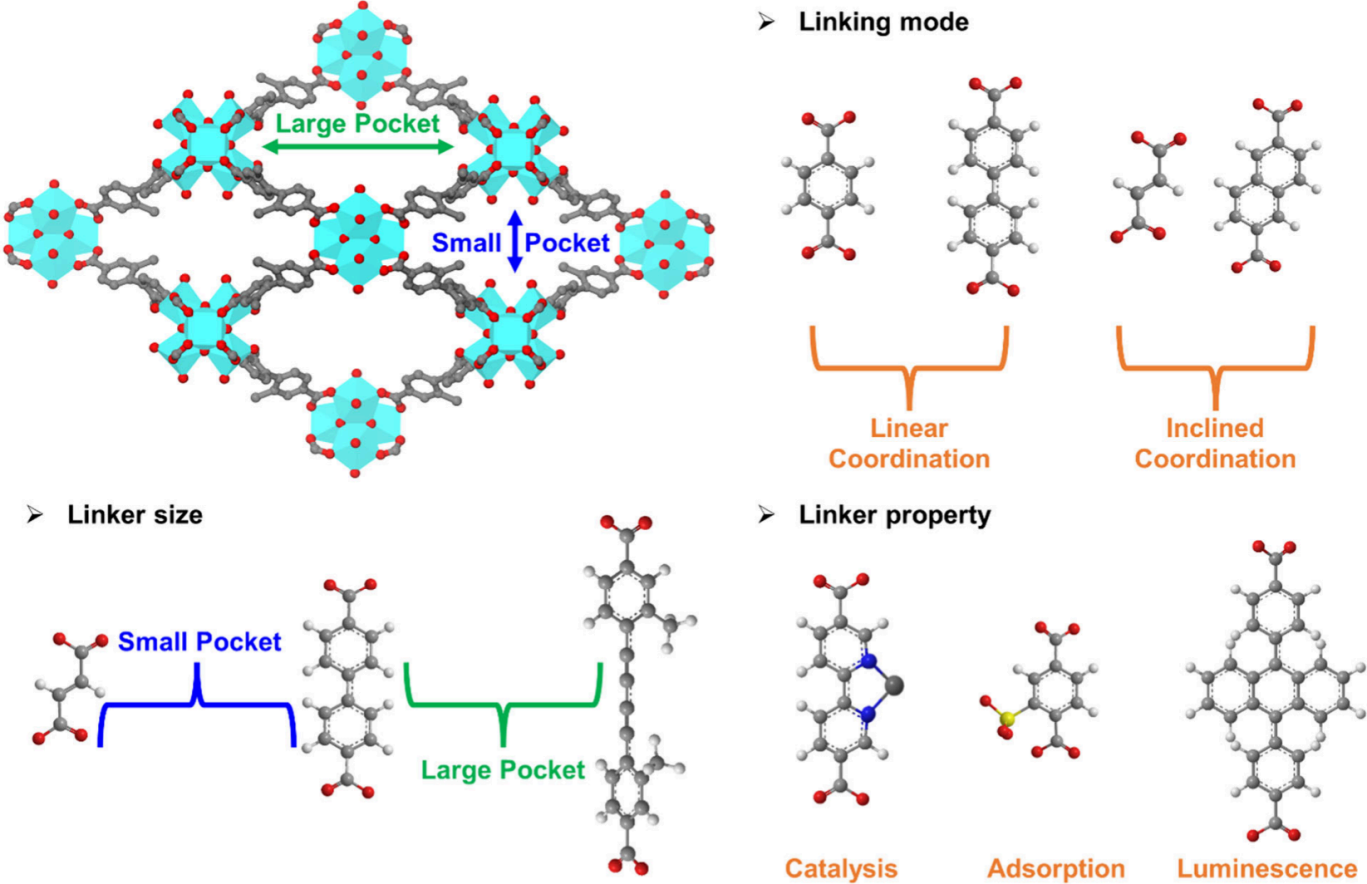
Linker selections for sequential linker installation based on PCN-700.

In order to implement the SLI process, the linkers must first be carefully selected. The selection process considers various factors, mainly including the size, linking mode, and property of the linkers (Figure 7).

For the linker size, given the flexible structure requirement discussed earlier, a range of ligands can be chosen. For example, in PCN-700, there are two types of missing linker pockets with lengths of 7.0 and 16.4 Å.^1^ Due to its adaptable framework, the smaller pocket can accommodate molecules ranging from about fumarate to biphenyl-4,4′-dicarboxylate. The larger pocket can fit molecules from about biphenyl-4,4′-dicarboxylate to 4,4′-(1,3-butadiyne-1,4-diyl)bis(3-methylbenzoate).^2^

For the linking mode, typically, linkers with a linear, 180° intersection angle, such as 1,4-benzenedicarboxylate and biphenyl-4,4′-dicarboxylate, are used frequently.^2^ However, for highly flexible structures like PCN-700, linkers with other intersection angles can also be installed, such as fumarate and 2,6-naphthalenedicarboxylate.^2^

For the property of the installed linker, the selection of the linkers focuses on enhancing the overall properties of the MOF, such as catalysis,^2^ adsorption,^3^ and luminescence.^45^ For example, in PCN-700, Cu-coordinated 2,2′-bipyridine-5,5′-dicarboxylate is selected for catalytic applications,^2^ 2-sulfoterephthalate for adsorption functions,^3^ and 4,4′-(anthracene-9,10-diyl)dibenzoate for enhanced luminescence properties.^45^ These specific linkers are selected to improve the desired functional attributes of the framework.

In addition to the selection of the appropriate linkers, other factors such as solvent choice, linker concentration, heating temperature, and heating duration also significantly influence the SLI process. For the solvent choice, the chosen solvent must effectively dissolve the installed linkers and maintain the stability of the selected MOFs. The solvent compatibility ensures that both the installed linker molecules and selected MOF structures remain intact during installation. For the linker concentration, the selected MOFs must remain stable at the concentrations of the installed linkers used. Maintaining this stability is crucial to prevent degradation or structural changes in the selected MOFs. For the heating temperature, both the installed linkers and the selected MOFs must withstand the heating temperature. Stability at this temperature is essential to preserve the integrity of the framework and ensure proper linker attachment. For the heating duration, extended heating time can increase the amount of linker incorporation but may also damage part of the selected MOFs. Therefore, optimizing the duration is critical to balance linker integration and framework preservation.

**Figure 8:**
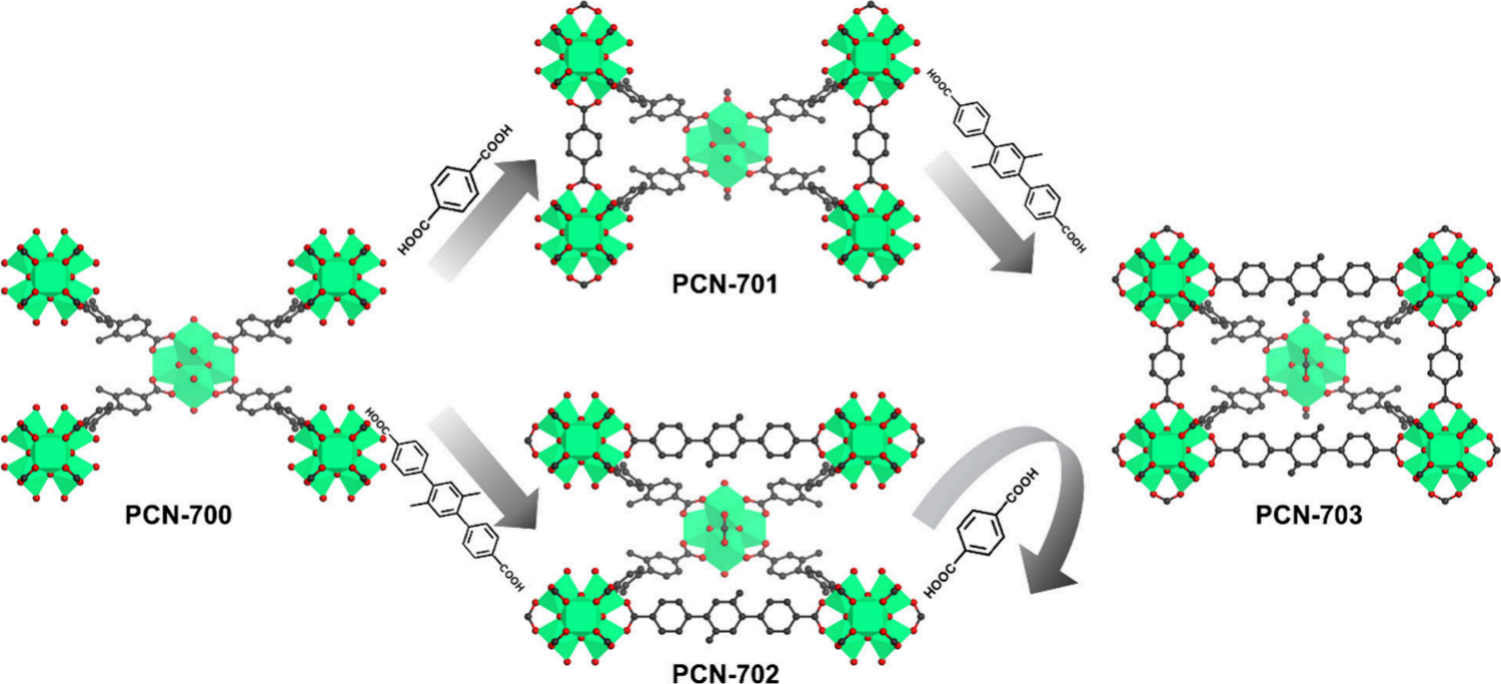
Linker installation sequence based for PCN-700.

Additionally, in some special cases, multiple linkers need to be installed into a single MOF, requiring careful design of the installation sequence. For example, PCN-700 possesses two types of missing linker pockets that can be installed with different linkers simultaneously to impart various properties. Our findings indicate that the shorter linker for the smaller pocket should be installed first, followed by the longer linker for the larger pocket. This sequence is essential because installing the linker in the larger pocket first significantly enhances the structural rigidity, thereby hindering the installation of the shorter linker in the smaller pocket (Figure 8).^1^

In summary, the SLI process in MOFs requires meticulous selection and optimization of various factors to achieve the desired material properties. The careful selection of linkers based on size, linking mode, and their specific property is fundamental, while the choice of solvent, linker concentration, heating temperature, and duration are also critical parameters that can obviously influence the success of the SLI process. Additionally, in cases requiring multiple linkers, the sequence of installation must be carefully designed to avoid structural rigidity issues that could hinder the process. By addressing these factors, the SLI process can be effectively implemented to create novel MOFs with tailored properties for a wide range of applications.

## 4 Enhanced Functions through the Installed Linkers

Installing linkers enables precise control over MOF structures, directly influencing factors such as pore size, pore shape, internal environment, framework rigidity, and structural stability. These enhanced properties significantly improve the functionalities of MOFs, particularly in catalysis, adsorption, and molecular recognition.

**Figure 9:**
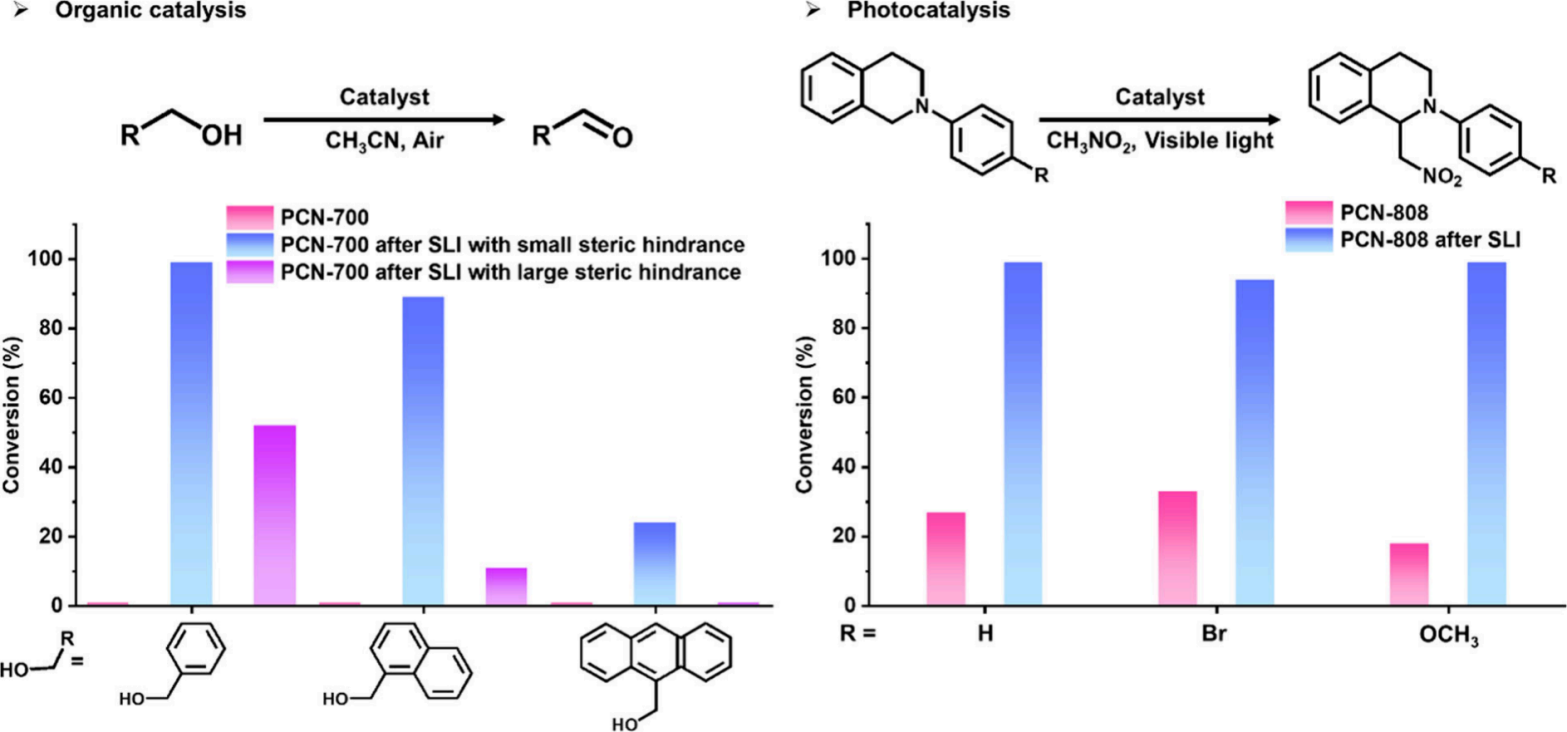
Enhanced catalysis functions by installed MOFs.

SLI in MOFs is a powerful approach to enhance their catalytic functions, by introducing specific functional groups or catalytic sites for various reactions. For traditional organic catalysis, Cu-coordinated 2,2′-bipyridine-5,5′-dicarboxylate was selected as the catalytic active center and terphenyl-4,4’’-dicarboxylate with different substituents were chosen to control the cavity size, to be installed into PCN-700, which work synergistically as a size-selective catalyst for aerobic alcohol oxidation.^2^ For photocatalysis, the photochemical active center bis(2,2′-bipyridine,*N*1,*N*1’)(5,5′-dicarboxy-2,2′-bipyridine-)ruthenium(II) was installed into PCN-808, which exhibited great photocatalysis activity and stability for the aza-Henry reaction and the oxidation of dihydroartemisinic acid to artemisinin (Figure 9).^44^

**Figure 10:**
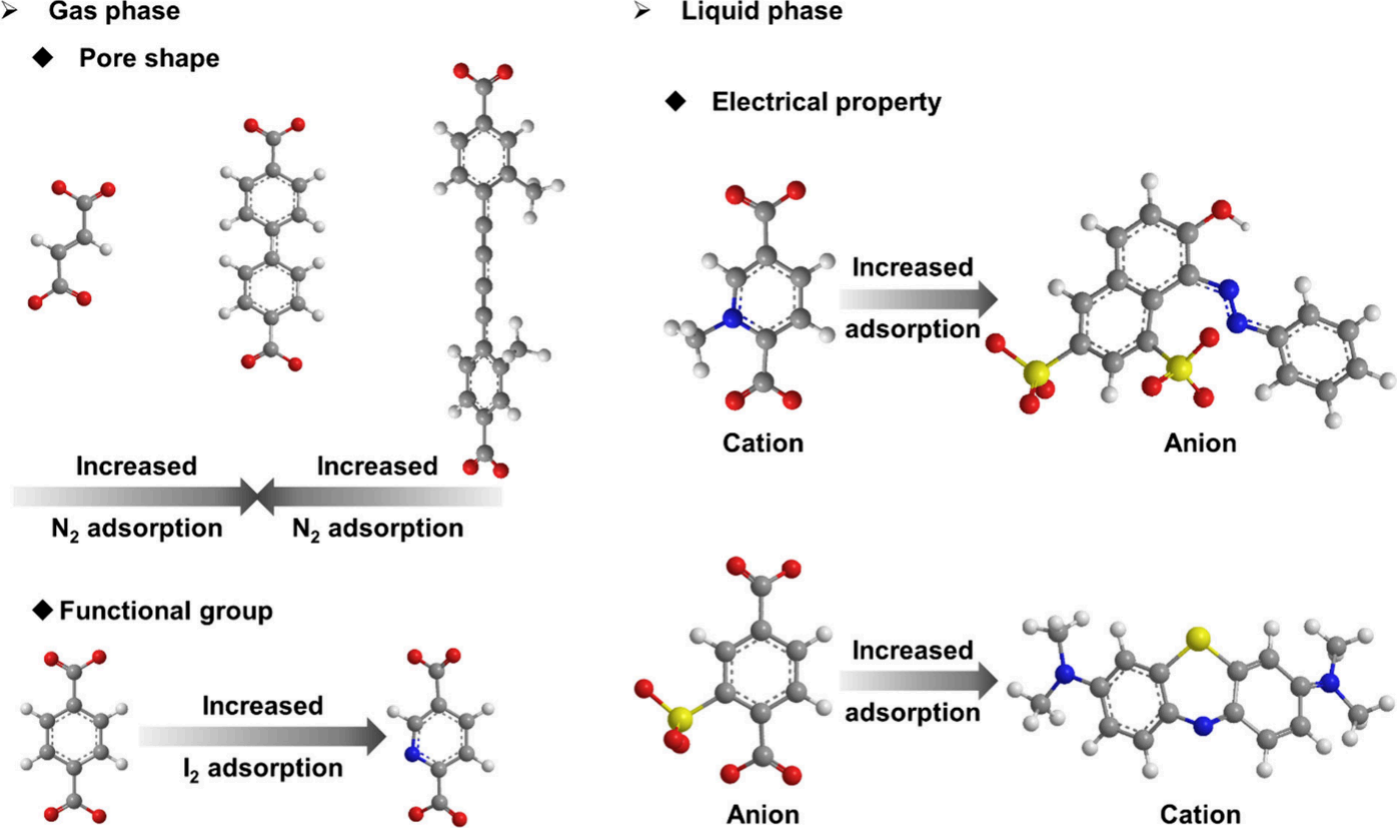
Enhanced adsorption functions by installed MOFs.

SLI in MOFs plays a crucial role in optimizing their adsorption capabilities. By carefully selecting and installing linkers with specific lengths, shapes, and functional groups, we can finely tune the internal surface chemistry of MOFs. These modifications significantly enhance the capacity and selectivity of MOFs in both gas and liquid phases. In the gas phase, SLI can influence gas adsorption abilities in several ways. Generally, the SLI process affects MOF porosity in two opposing manners. On the one hand, the installed linker occupies the free space inside the MOF cavity, decreasing the porosity. On the other hand, a linker with an appropriate length can open up the MOF cavity, increasing the porosity. These effects can lead to increased or decreased gas adsorption properties, as observed in PCN-700 with different installed linkers.^2^ Additionally, functional groups on the linkers can enhance adsorption capacities toward specific guests through intermolecular forces. For example, the installed 2,5-pyridinedicarboxylate has shown increased iodine vapor adsorption compared to the primary Th-MOF and the installed 1,4-benzenedicarboxylate.^53^ In the liquid phase, installing ionic linkers can significantly improve the selective capture of ionic dyes with heterogeneous charge. For instance, the adsorption of Orange G anions is greatly increased by the installed 2,5-dicarboxy-1-methyl-pyridin-1-ium cations in PCN-700 and PCN-608. Similarly, the adsorption of methylene blue cations is substantially enhanced by the installed 2-sulfoterephthalate anions in these MOFs (Figure 10).^3^

**Figure 11:**
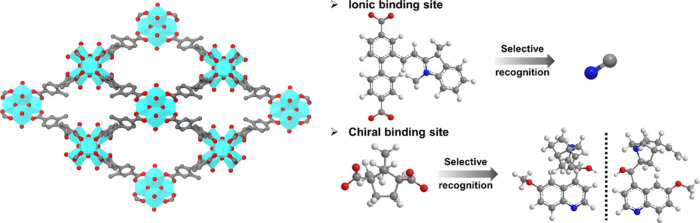
Enhanced recognition performance by installed MOFs.

SLI in MOFs can significantly enhance luminescence sensing functions by enabling precise manipulation of the MOF’s internal environment and introducing functional groups that improve luminescence properties and interact specifically with target molecules. For example, we first installed 4,4′-(anthracene-9,10-diyl)dibenzoate as the luminescence center into PCN-700. Subsequently, we incorporated 2-formyl-1,1′-biphenyl-4,4′-dicarboxylate and introduced 1,2,3,3-tetramethyl-3*H*-indolium iodide on the 2-formyl-1,1′-biphenyl-4,4′-dicarboxylate to serve as ionic binding sites. The resulting MOF exhibited a turn-on selective response toward CN^–^ due to its binding with the installed linker and a synergistic luminescence effect.^45^ Additionally, we installed the chiral center _D_-camphorate into PCN-700, allowing the ligand of PCN-700 to serve as the luminescence center. This construction, referred to as PCN-700-C, demonstrated enantioselective luminescent sensing properties toward a series of chiral drugs (Figure 11).^4^

Overall, SLI in MOFs provides precise control over their structural properties, pore environment, and structural stability, significantly enhancing their functional capabilities, particularly in catalysis, adsorption, and molecular recognition. The advantages of the SLI method, including structural maintenance, structural determination, and highly designable structures and properties, make it an excellent candidate for the functional-guided design and synthesis of MOFs.

## 5 Conclusion and Outlook

In conclusion, the development of the SLI method for MOFs has significantly advanced the field of porous materials. Through innovative techniques, researchers have overcome challenges related to linker incorporation, enabling precise control over MOF structures and influencing their properties including catalysis, adsorption, and luminescence. Besides, the exploration of novel combinations of suitable MOFs and installed linkers holds immense promise for further enhancing the versatility and performance of MOFs.

Future research on SLI methods may primarily focus on scalable synthesis, novel properties, and practical applications. In synthesis, the development of scalable and cost-effective techniques to improve the efficiency of the SLI process for MOFs with tailored properties remains challenging. Key issues include the excessive use of linkers and solvents, achieving large-scale production, and managing energy consumption. For novel properties, functional organic molecules with coordination units like carboxyl and hydroxyl groups must be incorporated at appropriate positions during installation, a task that is often complex. For practical applications, molding powder samples into devices such as blocks or membranes may present additional challenges for the installation process.

In summary, SLI method represents a cornerstone in the continued evolution of MOF research, offering exciting opportunities for the design and fabrication of next-generation porous materials with unprecedented properties and functionalities. As interdisciplinary collaborations continue to flourish and technological advancements accelerate, the future of MOF science appears exceptionally bright.
